# Do Environmental Prompts Work the Same for Everyone? A Test of Environmental Attitudes as a Moderator

**DOI:** 10.3389/fpsyg.2019.03057

**Published:** 2020-02-05

**Authors:** Lisa Selma Moussaoui, Olivier Desrichard, Taciano L. Milfont

**Affiliations:** ^1^Faculty of Psychology and Educational Sciences, Université de Genève, Geneva, Switzerland; ^2^School of Psychology, Victoria University of Wellington, Wellington, New Zealand

**Keywords:** person-situation interaction, environmental concern, attitude, prompt, cues, pro-environmental behavior

## Abstract

The extant literature has focused either on personal variables or on situational factors to explain pro-environmental behavior despite several calls to integrate both. The present research addresses this integration call by testing the interaction between environmental attitudes and situational prompts on pro-environmental behavior. Three experimental studies manipulate the presence/absence of pro-environmental prompts, measure environmental attitudes, and investigate the effect of both variables on behavior. Study 1 showed a simple effect: participants with higher levels of pro-environmental attitudes (compared to those with lower levels) performed more energy saving behavior in the presence of prompts. However, in the absence of prompt, none of the participants performed the behavior, which prevented us from statistically testing the interaction. Studies 2 and 3 were conducted with a similar design: main effects of attitude and prompts were obtained, but the interaction was not found. A Bayesian analysis of the data suggested more evidence toward the null hypothesis of no interaction between environmental attitudes and situational prompts.

## Introduction

The majority of people in 78 countries support environmental protection (Milfont and Schultz, 2016), and a recent large-scale survey across 28 European countries also found that 94% of respondents express that protecting the environment is “very or fairly important” (European Commission, 2017). This widespread support for environmental protection is contrasted by low actual pro-environmental behavior. Indeed, the same European survey showed that behaviors, such as choosing an environmentally friendly travel transport mode, choosing local products, reducing energy consumption, or avoiding single-use plastic goods (other than plastic bags), are endorsed by only a third of the respondents. This indicates that around two-thirds of people who express being in favor of environmental protection do not perform pro-environmental behavior in their daily life. Other international surveys conducted in Brazil, Canada, China, India, the USA, and some European countries have reported similar results (Bonini et al., 2008), suggesting that this attitude-behavior gap is widely spread.

According to Schultz et al. (1995), the extant literature has focused on two types of variables explaining pro-environmental behavior: (1) personal variables, such as attitudes, knowledge, personality, and demographic variables and (2) situational factors, such as prompting, commitment, feedback, normative influence, rewards, and removing barriers. These authors suggested that research should look at interactions between those two types of variables instead of studying them separately (see also Guagnano et al., 1995). Although the recommendation to explore the interaction between personal and situational variables was published more than 20 years ago, we could locate only a few published studies integrating personal variables and situational factors (see, for example, Taube and Vetter, 2019 for the same question on defaults), and none of them tested the interaction of environmental attitudes and prompts on subsequent ecological behavior. The present study addresses this gap by examining the extent to which individuals’ environmental attitudes interact with situational prompts to induce energy-saving behavior.

The association between attitudes and behavior has been studied in several domains of psychology (for reviews, see Bohner and Dickel, 2011; Albarracin and Shavitt, 2018; Milfont and Schultz, 2018). Although scholars have argued that attitudes are often only weakly related to behavior (e.g., Wicker, 1969), meta-analytical reviews have suggested that both implicit and explicit measures of attitudes are reliably correlated with attitude-relevant behaviors (Glasman and Albarracin, 2006; Greenwald et al., 2009). Similarly, the association between environmental attitudes and behavior has been shown to be low to moderate (Bamberg, 2003; Gupta and Ogden, 2006), but meta-analytical reviews indicate that environmental attitudes are reliably correlated with behavioral intentions and behavior (Bamberg and Moser, 2007; Milfont, 2012; Morren and Grinstein, 2016).

Authors have discussed reasons for the lack of a strong correspondence between attitudes and behavior, including theoretical and methodological aspects (see, for example, Weigel and Newman, 1976; Ajzen and Fishbein, 1977; Kaiser et al., 1999; Bamberg, 2003). More important to the present study is Wicker (1969), who suggested that situational factors could improve attitude-behavior consistency. In the same vein, Triandis (1977) argued that facilitating conditions in the environment play a role in determining behavior when habits are in place.

A type of situational factor that has been shown to successfully promote pro-environmental behaviors is prompts, or reminders, of when to perform a specific action (Osbaldiston and Schott, 2012). In their meta-analysis of 44 publications using prompts, Osbaldiston and Schott (2012) reported a moderate-to-high effect size for prompts in promoting pro-environmental behavior (*g* = 0.62). They also reported variation between domains, with a moderate effect of prompts for public energy conservation (*g* = 0.54) and a high effect for public recycling (*g* = 0.95). Schultz (2014) stated that prompts work best for easy and repetitive behavior, and when prompts are displayed directly in the context where the person has to act. This type of prompt is often called a “point-of-decision prompt” because the prompt is displayed at the moment and place the person has to choose to perform or not perform the behavior. The present study used “point-of-decision prompts” to induce pro-environmental behavior. For simplicity, we will use the term prompts.

Schultz (2014) argues that prompts are effective because, in some cases, individuals simply forget to act, and the prompt functions as a reminder. This implies that individuals are motivated to act but might get distracted, or the appropriate pro-environmental behavior is not salient. A person might not act pro-environmentally in a given situation despite holding pro-environmental attitudes because he or she simply does not know how or when to act or even whether they should act at all. For example, imagine someone entering a public toilet in which the light is on. In absence of a prompt asking people to turn off the light when they leave, this person might think that the light is automatic, inferring this is why it was on when they arrived, and that it will turn off automatically after a certain time. In this situation, the absence of a prompt might lead the person to miss an opportunity to act in accordance with held beliefs.

Thus, the person-situation interaction, a classical question in the field of personality study (Schmitt et al., 2013), seems to be an appropriate framework for the question of the interaction between environmental attitudes and prompts. Theoretical discussion of this question can be traced back, at least, to Lewin’s (1951) person × situation analysis. It proposed that the behavior is a function of both personal and situational variables: the impact of personal variables on behavior depends on the situation the person is in, and, vice versa, the impact of the situational variables does not influence everybody in the same way. Personal variables in this model include attitudes among other variables, such as traits and emotions, and situational variables encompass physical and social environments (Kihlstrom, 2013).

To the best of our knowledge, no previous empirical study has directly tested the predicted interaction between environmental attitudes and prompts for triggering pro-environmental behavior. Nevertheless, some studies testing related research questions were located. Bolderdijk et al. (2013) examined the interaction between informational interventions and values. They showed that a video about the negative environmental consequences of using bottled water had an impact on pro-environmental intention only for participants who held biospheric environmental values. A study by Wiese et al. (2004) explicitly tested the interaction between environmental concern and the presence of prompts on the probability to read instruction manuals for electrical consumer products. Levels of environmental concern influenced the probability to read instructions on how to use the device in an energy-saving way, but that was the case only in the presence of a prompt pertaining to ecology. However, the main target of this research was to study how people use written product information, which explains why the dependent variable was not a direct environmental behavior. Other studies focused on the same concepts but did not tested the interaction of interest. For example, Kurz et al.’s (2005) intervention study measured environmental attitudes and used prompts (named attunement labels in their paper), but the authors examined whether attitudes were correlated with water consumption at baseline and not whether attitudes interacted with prompts in predicting water consumption. Unrelated to prompts but following the same reasoning for smart-meter-based feedback interventions, Henn et al. (2019) argued that “a certain level of motivation apparent in a person’s environmental attitudes is required for feedback to become effective” (p. 75).

Similarly, it is fair to expect that prompts do not have any behavioral impact unless a certain level of environmental concern is present. Said differently, we could imagine that an individual with high levels of environmental attitudes would have been inclined toward carrying out environment-friendly behavior but would need a prompt as a reminder or trigger (for reasons of ambiguity, habit, forgetfulness, etc.), while another individual with low levels of environmental attitudes would not carry out the environment-friendly action even in the presence of a prompt and even if the behavior was very clear and the person was not constrained by habits. The present research describes three experiments testing the environmental attitude-prompt interaction. Main effects for environmental attitudes and prompts in influencing pro-environmental behavior were predicted based on theoretical and empirical grounds. The novelty of this research was its testing of a *moderation hypothesis*: attitudes could influence individuals’ reaction to the environmental prompt, with participants reporting higher levels of pro-environmental attitudes being more sensitive to the prompts. To test these predictions, we designed two-part experiments allowing us to measure the environmental attitudes of participants and unrelatedly observe their reaction to prompts.

## Study 1

### Methods

#### Materials and Procedure

The experiment was conducted as part of a larger and unrelated survey project. No conditions or measures were dropped from the experiment. Participants completed two parts of a study that took place 1 week apart. The experimental manipulation was conducted in the first part. Participants were invited to come to a computer lab to complete Part 1 on individual computers. This consisted of an online survey comprising unrelated individual differences measures and background items, which gave us the opportunity to observe their energy-saving behavior: turning off or not turning off their computer screen once they have completed the survey. The survey questions had no link to the environmental topic to avoid confounding effects on the target behavior.

The computer lab was a square room with 15 computers organized in a u-shape form along the sides of the walls. All computers were isolated from each other by partition, and participants sat facing the computer screen and adjacent wall. Upon arrival, participants gave their student ID to the experimenter, which was used to match data between the two study parts and behavioral data, and they were asked to freely choose a computer to complete the study. A concluding message appeared on the screen at the end of the survey thanking the participants for completing the survey and reminding them that they would receive an email to complete Part 2 of the study 1 week later. The concluding message differed across conditions and was our experimental manipulation, with participants in the prompt conditions also asked to turn off the computer screen. The web-based survey automatically and randomly allocated participants either to the control condition or to one of three prompt conditions described below.

Past research has indicated that the level of the behavioral goal described in prompt messages might impact behavior differentially (Moussaoui and Desrichard, 2016). Following this past research, we used three distinct types of messages in each of the prompt conditions. We added concluding messages for each prompt condition: “Press on the button to turn off the computer screen” (message-only prompt); “Press on the button to turn off the computer screen, in order to lower energy use at Victoria University of Wellington” (low-level goal prompt); and “Press on the button to turn off the computer screen, in order to preserve resources of the planet” (high-level goal prompt). The Supplementary Materials present the specific prompts used. This variety in the material is illustrative of the various types of prompts used in pro-environmental campaigns. Participants in the control condition were not exposed to any prompt or message requesting them to turn off the computer screen. The experimenter discreetly recorded the behavior of each participants (i.e., turning off or not turning off the screen). This dependent variable was dummy coded (0 = did NOT turn off computer screen, 1 = did turn off computer screen).

One week after completing the first part of the study, all participants received an email with a link to complete the online survey for Part 2. This second phase did not take place in the lab. Participants could complete the online survey in their own time and in any computer with internet. Participants first completed a series of individual differences measures, including the environmental attitudes measure detailed below. After this randomized block of measures, participants then answered questions about the experimental manipulation. First, we used a recall question: “The following question is related to the moment you did the first part of the experiment, in room (number of the room). For some computers, a message appeared at the end of the study. Do you remember what was written in this message?” This question was presented to all participants and allowed us to examine whether participants remembered a message was presented to them or not and also whether they remembered what the message contained. The response options were the three versions of the prompts, plus “No such message appeared at the end of the study,” and “I don’t remember.” Once the allotted time to complete Part 2 of the study was expired, all participants received an email with a full description of the research goals and debrief.

#### Participants

A total of 47 students took part in a pilot phase of the study used to finalize the prompts presentation and study layout. After that, a total of 191 first-year psychology students from the Victoria University of Wellington, New Zealand, enrolled to take part in the study for partial fulfillment of a course requirement, and 185 completed both parts of the study (i.e., a 3% drop out, all in the prompt condition). The sample size was based on the availability of participants in the students’ pool. This sample comprised 144 women and 41 men with a mean age of 18.73 years (SD = 2.31).

#### Measures

As mentioned above, we conducted the experiment as part of a larger and unrelated survey project. The Part 1 survey included five individual difference measures: the Basic Value Survey (Gouveia et al., 2014), the Need for Closure Scale (Webster and Kruglanski, 1994), the Brief Self-Control Scale (Tangney et al., 2004), the Positive and Negative Affect Schedule (Watson et al., 1988), and the Self-Construal Scale (Vignoles et al., 2016). The Part 2 survey included the Brief Self-Control Scale again and four other measures not considered here: outcome expectancy and cumulative effort (Moussaoui and Desrichard, 2016), the Consideration of Future Consequences scale (Joireman et al., 2012), and Behavior Identification Form (Vallacher and Wegner, 1989), plus background questions similar to those used in Part 1 and the measure of environmental attitudes considered in the present research.

The key measure in the Part 2 survey was the instrument assessing the participants’ environmental attitudes. We used the 24-item version of the Environmental Attitudes Inventory (EAI-24) (Milfont and Duckitt, 2010) that measured beliefs about environment Preservation (e.g., “Humans are severely abusing the environment”) and Utilization (e.g., “I DO NOT believe humans were created or evolved to dominate the rest of nature”). Items were rated from 1 (*strongly disagree*) to 7 (*strongly agree*). The overall EAI score showed satisfactory reliability (Cronbach’s *α* = 0.851; *M* = 4.74, SD = 0.64) as well as each sub-dimension of the scale (Preservation: Cronbach’s *α* = 0.825; Utilization: Cronbach’s *α* = 0.706).

### Results

#### Preliminary Results

We used the recall question to provide an indication of participants’ recall of the message they were exposed to. Among participants in the control condition, 65% were certain that no message appeared at the end of the study, 26% said they did not remember, and 9% wrongly assumed that they noticed a message. For participants in the experimental condition, 4% said wrongly that no message appeared, 11% did not remember, and the other 86% indicated that they had seen one of the three prompts. Between 58 and 76% of them remembered the correct version of the message they saw at the end of Part 1 survey. Those results confirm that participants read the prompts. Recall is very high considering that participants were presented with the message 1 week before and that participants did not know they had to remember this information.

We also examined whether the three versions of the prompts had distinct main effects on behavior. A logistic regression was conducted with the prompt type predicting energy-saving behavior, and the results were not statistically significant: Wald = 0.008, *p = 0*.927, OR = 1.05, 95% CI (0.37, 2.97) for low-level goal prompt vs. message-only prompt, and Wald =1.44, *p* = 0.230, OR = 2.04, 95% CI (0.64, 6.54) for high-level goal prompt vs. message-only prompt. This result indicated that there was no variability across the prompt type. We then combined all three prompt conditions in a single experimental prompt condition to compare with the control condition in the main analyses.

#### Main Results

**Figure 1:**
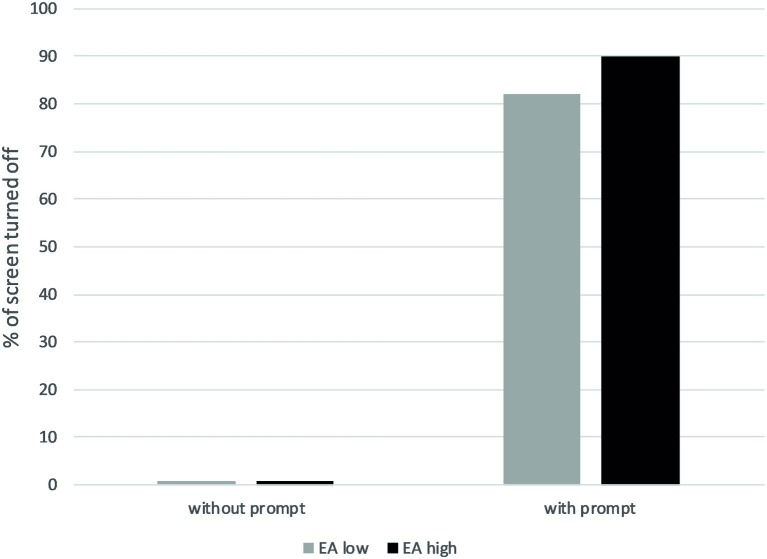
Percentage of participants turning off their computer screen according to the presence/absence of prompts and their level of environmental attitudes (median split) in Study 1.

The percentages of screens turned off according to conditions and level of environmental attitudes are presented in Figure 1. Our two *main effects* hypotheses predicted that behavior would depend on the presence of prompts and participants’ environmental attitudes. Frequency tables showed that, in the control group, none of the 31 participants did turn off the screen, whereas when the prompt was present a total of 138 of 160 participants (86.25%) did turn off the screen. This clearly supports the main effect of prompts on energy-savings behavior.

The association between environmental attitudes and behavior was tested with a logistic regression. Since there was no variability in behavior in the control group, we tested this association only in the experimental group. The association between environmental attitudes and behavior was significant, Wald = 5.00, *p* = 0.025, OR = 2.44, 95% CI (1.12, 5.34), meaning that participants in the experimental group with higher levels of environmental attitudes were more likely to turn off the computer screen. For ease of comparison across studies, effect sizes were converted to Cohen’s *d*. The OR for the association between environmental attitudes on behavior obtained in Study 1 corresponded to a *d* = 0.49. We conducted the same logistic regression this time with the Preservation and Utilization scores separately instead of the general EAI score. The Preservation dimension was significantly related to behavior, Wald = 6.69, *p* = 0.010, OR = 2.53, 95% CI (1.25, 5.13), *d* = 0.51, while the Utilization dimension was not, Wald = 1.44, *p* = 0.231, OR = 0.679, 95% CI (0.361, 1.28), *d* = −0.21.

Because of the lack of variability in the control group, we could not statistically test our interaction hypothesis. Thus, our research question was not answered in the Study 1 results.

### Discussion

In this first study, we showed that participants with higher levels of pro-environmental attitudes behaved in a more environment-friendly way than participants with lower levels of pro-environmental attitudes when a prompt was present to remind them to act. We also showed that, globally, participants tended to be more pro-environmental when a prompt was present than when it was not.

The fact that no participant in the control condition turned off the computer screen was unexpected, and suggested that, in the absence of prompts, it is possible that participants might have thought they had to leave the screen on. On one hand, this argues in favor of the utility of prompts, which renders situations clearer, unambiguous, and make the pro-environmental option more salient. On the other hand, this constitutes an alternative explanation that would call the existence of the interaction into question. This explanation is plausible given the experimental situation where participants might have thought the experimenter would need the computer screen to be left on. To rule out this possibility, we designed a second study with no such ambiguity for participants.

## Study 2

### Methods

#### Materials and Procedure

The experiment was designed in the same format as Study 1, but the behavioral measure was modified in order to be explicit for participants that they had to take an action. Participants were invited to come to a computer lab to complete the first part on individual computers. They had to complete an unrelated task on the computer (a pilot study for another unrelated project: listening to sounds and rate the sounds on several variables). Then, they were asked to print the consent form, and they had to choose settings for the print: orientation (portrait vs. landscape); color vs. black/grayscale; 2-sided printing; quality (superior, fine, draft); paper type (normal vs. recycled). Color vs. black/grayscale and quality choices influenced the amount of ink used for printing. All of these choices except Orientation (added to increase the ecological validity of the task), constituted our behavioral dependent variable [sum score ranging from 0 for the least environment-friendly choices to a maximum 4 if the person choose all the most environment-friendly options (i.e., black/grayscale, 2-sided printing, draft quality, recycled paper)]. On the Printing Preferences window, an environmental prompt appeared in the “prompt” condition, asking participants to *Please consider the environment while printing*, while no environmental appeal appeared in the “no-prompt” condition (see Supplementary Materials for screenshots). Then participants were informed that the session was over and were thanked for their participation. A text entry box was available if they had any comment about the experiment. As in Study 1, 1 week after completing the first part of the study, participants received an email with a link to complete the second part online, which measured environmental attitudes, a question on recall of the message, and study debriefing.

#### Participants

A total of 150 second-year psychology students from Geneva University, Switzerland, took part in both parts of Study 2 for partial fulfillment of a course requirement. A total of 161 students completed Part 1, and 11 dropped out (6.8%) – three from the “prompt” condition, and eight in the “no-prompt” condition. Sample size was based on the availability of participants in the students’ pool. Eight participants were excluded because they expressed suspicion about the printing task in the text entry box at the end of the first session (for more detailed justification of this choice, see Supplementary Materials), resulting in a sample of 142, composed by 120 women and 22 men, with a mean age of 22.75 years (SD = 5.08).

#### Measures

Environmental attitudes were measured again with the 24-item version of the Environmental Attitudes Inventory (EAI-24) but this time in French (Moussaoui et al., 2016). Similarly, as in Study 2, items were rated from 1 (*strongly disagree*) to 7 (*strongly agree*). The EAI validated in French showed satisfactory reliability (Cronbach’s *α* = 0.772; *M* = 4.89, SD = 0.57). The reliability of each sub-scale was lower than for the full scale (Preservation: Cronbach’s *α* = 0.693; Utilization: Cronbach’s *α* = 0.608).

The recall item consisted of asking participants which message they had seen the preceding week on the printing preferences page. Four screenshots were presented: “Please consider the environment while printing” (the correct answer for participants in the prompt condition); “Please make your printing choices below” (the correct answer for participants in the no-prompt condition); “Please save paper while printing” (filler); no message (filler); and a radio button “I don’t remember.”

### Results

#### Recall Rate

One week after the first session, 44.4% of participants in the prompt condition recognized the correct message, while 2.9% of participants in the no-prompt condition erroneously said they had seen the same prompt (“Please consider the environment while printing”). The majority of participants (61.4%) in the no-prompt condition said they had seen the sentence “Please make your printing choices below,” which was correct, while 31.9% of participants in the prompt condition said wrongly they had seen this message too. A small number of participants in the prompt condition (4.2%) and no-one in the no-prompt condition said they saw the filler prompt (“Please save paper while printing”). Around 6% in each condition said there was no message, and 12.5% in the prompt condition and 30% in the no-prompt condition answered they did not remember.

#### Main Results

**Figure 2:**
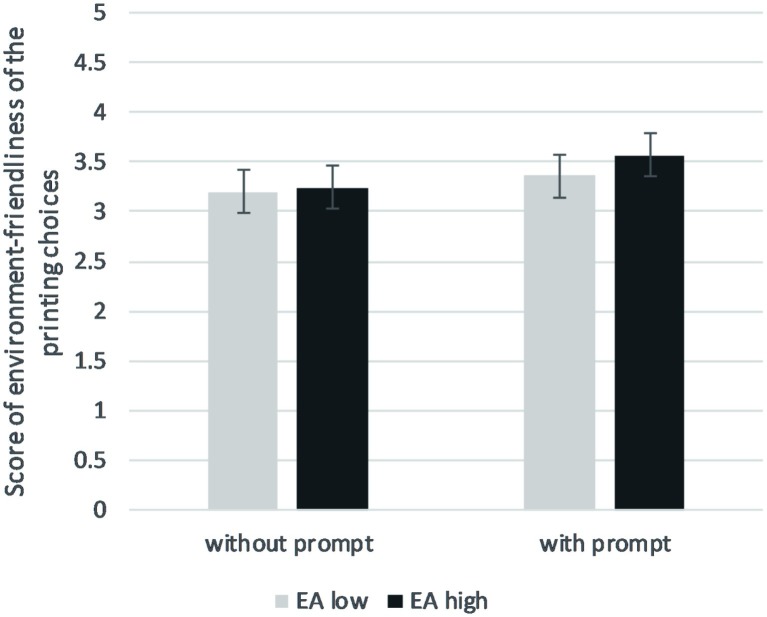
Score of printing choices (highest = more environment friendly) according to the presence/absence of prompts and the level of environmental attitudes (median split) in Study 2. Errors bars represent 95% CI.

An ANOVA was used to test our main effects and interaction hypothesis. Means according to the experimental condition and the level of attitudes are presented in Figure 2. The association between environmental attitudes and behavior was marginally significant, *F*(1, 138) = 3.13, *p* = 0.079, $\eta_{p}^{2}$ = 0.022, meaning that there was a trend for participants with higher levels of environmental attitudes to select environment-friendly options when printing (Cohen’s *d* = 0.30). The main effect of prompts was statistically significant, *F*(1, 138) = 6.93, *p* = 0.009, $\eta_{p}^{2}$ = 0.048: printing choices were more environment-friendly in the presence of prompts (Cohen’s *d* = 0.45). However, no interaction appeared between environmental attitudes and prompts, *F*(1, 138) = 0.58, *p* = 0.449, $\eta_{p}^{2}$ = 0.004. Similar to Study 1, we ran the analysis on the two sub-dimensions of attitudes. Neither the main effect of Preservation on behavior, *F*(1, 138) = 2.14, *p* = 0.146, $\eta_{p}^{2}$ = 0.015, nor the interaction with the prompt condition, *F*(1, 138) = 1.05, *p* = 0.306, $\eta_{p}^{2}$ = 0.008, was significant. Similar results were obtained for Utilization: main effect *F*(1, 138) = 2.21, *p* = 0.140, $\eta_{p}^{2}$ = 0.016 and the interaction, *F*(1, 138) = 0.00, *p* = 0.974, $\eta_{p}^{2}$ = 0.000.

Because a non-significant *p* value cannot be interpreted as proof of non-existence of the effect (Dienes, 2014), Bayesian statistics were used in order to provide more insight into the possible lack of interaction. Indeed, the non-significance of the interaction obtained in Study 2 could either be caused by data insensitivity or be an indicator of no interaction among our variables of interest. We used the model comparison procedure (using a uniform prior) to test the likelihood of a model containing only main effects (the null model) compared to a model including the interaction. The model comparison suggested that the data were slightly more likely to be observed under the null model containing no interaction, as confirmed by a Bayes factor of BF_01_ = 2.683, meaning the data were close to three times more likely to have occurred under the null hypothesis (only main effects) than under the alternative hypothesis (including interaction). However, given the size of the Bayes factor, strength of evidence would be considered as anecdotal (Jeffreys, 1961; Lee and Wagenmakers, 2013).

### Discussion

Results from Study 2 confirmed a main effect of prompts and a marginal effect of environmental attitudes but no significant interaction when selecting environment-friendly options when setting printing preferences. The dependent variable used in the second study was successful at removing the ambiguity issue that arose in the first study, and all participants made a choice, even in the no prompt condition. This might explain why the pattern was different from Study 1 where no participant turned off the screen when there was no prompt, even those with high levels of environmental attitudes, and argues in favor of the idea that the interaction obtained in Study 1 was an artifact. However, the size of the BF obtained in Study 2 is not sufficient to claim that no interaction happened. Consequently, we found that a third study was necessary in order to gain more support for one hypothesis or the other. Because the dependent variable in the second study was detected by some participants to be part of the experiment as reflected in the text entry box, we decided to change our dependent variable measure and use a less costly design with hypothetical choices.

## Study 3

### Methods

#### Materials and Procedure

The first part of the experiment was an online survey. Participants first completed a questionnaire for an unrelated project on hand hygiene. Then, the task for this study was presented: participants had to choose between two modes of transportation for a hypothetical trip. The experimental manipulation of the prompt consisted of a small green leaf logo appearing or not next to the mode of transportation considered as the most ecological. The presence/absence of prompt variable was manipulated in a within-participants design: of the six choices, two had the prompts, two did not, and two were fillers in order to mask the research objective (see Supplemental Materials for more details on the scenarios). Which choices had the prompts or not was presented randomly across participants^1^ and controlled as block effect in the analysis. Participants were then informed that the session was over and thanked. A text entry box was available if they had any comment about the experiment.

As in Studies 1 and 2, 1 week after completing the first part of the study, participants received an email with a link to complete the second part online, which measured environmental attitudes, and the debriefing.

#### Participants

A total of 182 second-year psychology students from Geneva University, Switzerland, participated in both parts of Study 3 for partial fulfillment of a course requirement (194 completed Part 1, 12 dropped out = 6.2%). Sample size was based on the availability of participants in the students’ pool. Two participants were excluded because they expressed suspicion about the prompt labels, resulting in a final sample of 180 participants (155 women and 25 men, with a mean age of 22.23 years, SD = 4.29).

#### Measures

Six hypothetical journeys were presented to participants, two fillers (answers to those items were not considered) and four choices of interest. For each journey, the DV was the choice of transportation mode: ecological (e.g., train) or non-ecological (e.g., plane, car). Environmental attitudes were measured again with the French version of the 24-item Environmental Attitudes Inventory (EAI-24) (Moussaoui et al., 2016) and showed satisfactory reliability for the full scale (Cronbach’s *α* = 0.855; *M* = 5.01, SD = 0.69) and each sub-dimension (Preservation: Cronbach’s *α* = 0.790; Utilization: Cronbach’s *α* = 0.682).

### Results

**Figure 3:**
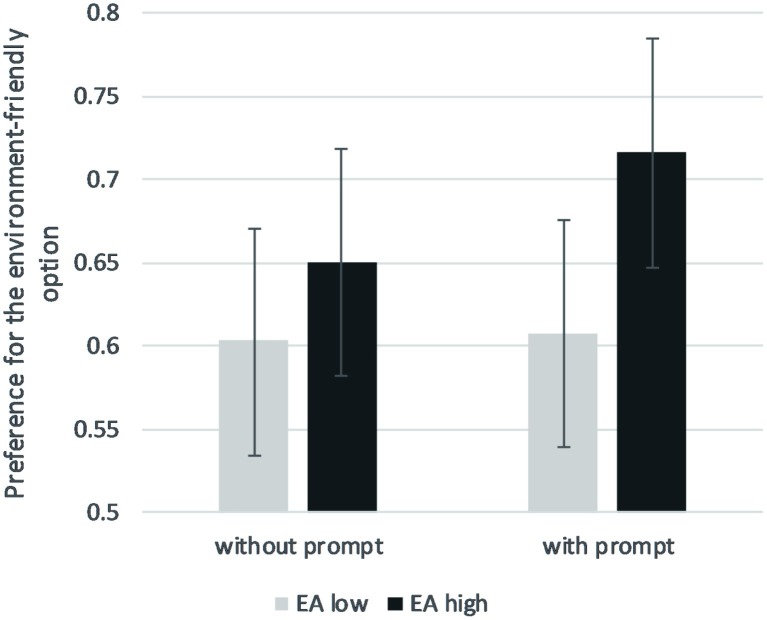
Preference score for the environment-friendly option according to the presence/absence of prompts and the level of environmental attitudes (median split) in Study 3. Errors bars represent 95% CI.

A repeated-measure ANOVA was used to test our main effects and interaction hypothesis. Means according to the presence or absence of prompts and the level of attitudes are presented in Figure 3. The association between environmental attitudes and behavioral choice was statistically significant, *F*(1, 173) = 15.88, *p* < 0.001, $\eta_{p}^{2}$ = 0.084. As expected, participants with higher levels of environmental attitudes were more likely to opt for the environment-friendly mode of transportation (Cohen’s *d* = 0.60). Unexpectedly, the main effect of the prompt did not reach statistical significance, *F*(1, 173) = 2.06, *p* = 0.153, $\eta_{p}^{2}$ = 0.012, but the direction of the effect was as expected (more environment-friendly choices when the prompt was present) (Cohen’s *d* = 0.22). Again, no interaction appeared between environmental attitudes and prompts on the dependent variable, *F*(1, 173) = 0.03, *p* = 0.863, $\eta_{p}^{2}$ = 0.000.

As for Studies 1 and 2, we ran the analysis on the two sub-dimensions of attitudes. The Preservation score was significantly associated with opting for the environment-friendly mode of transportation, *F*(1, 173) = 16.38, *p* < 0.001, $\eta_{p}^{2}$ = 0.086, *d* = 0.61, while the relationship was negative for the Utilization score, *F*(1, 173) = 9.03, *p* = 0.003, $\eta_{p}^{2}$ = 0.050, *d* = −0.46, and with a smaller effect size. In both cases, the interactions were not statistically significant: for Preservation, *F*(1, 173) = 0.08, *p* = 0.767, $\eta_{p}^{2}$ = 0.001, and for Utilization, *F*(1, 173) = 0.61, *p* = 0.436, $\eta_{p}^{2}$ = 0.004.

In order to counterbalance for which hypothetical journey the prompt was presented, we added the variable bloc as a control in the analysis. The bloc did not had a significant main effect, *F*(1, 173) = 0.77, *p* = 0.575, $\eta_{p}^{2}$ = 0.022, but interacted with the effect of the prompt, *F*(5, 173) = 11.04, *p* < 0.001, $\eta_{p}^{2}$ = 0.242. This interaction indicated that the effect of the prompt differed depending on the destination of the journey. For example, when the journey to Venice was combined with the presence of prompt and the journey to Paris with the absence of prompt (blocs 1 and 5), the effect was reversed compared to when Paris was associated with the prompt and Venice with no prompt (blocs 2 and 3)^2^. This was unexpected and might explain the non-significance of the main effect of the prompt. For the interested reader, we provide a more thorough description of this result in Supplementary Materials.

As for Study 2, we conducted a Bayesian model comparison (with uniform prior) to have more conclusive evidence regarding the lack of a statistically significant interaction. The Bayes factor of BF_01_ = 6.652 revealed that the data were over six times more likely to have occurred under the null hypothesis (model with only main effects) than under the alternative hypothesis (model including an interaction between attitudes and prompts). The strength of evidence would be considered as substantial (Jeffreys, 1961; Lee and Wagenmakers, 2013).

### Discussion

Results from Study 3 confirmed a positive association with environmental attitudes and non-significant effect of prompts (albeit descriptively in the hypothesized direction), but no significant interaction, when selecting environment-friendly travel mode choices. Notwithstanding differences in method (repeated measures instead of between-subject design) and the type of dependent variable (hypothetical choice instead of behavioral observation), the interaction did not occur in either Study 2 or Study 3. The size of the Bayes factor gives more confidence into the idea that there might not be an interaction between environmental attitudes and presence or absence of prompts. This is examined in more detail in the General Discussion.

An element of variation across studies that might be considered as a limitation is that, in Study 3, the prompts differed on two aspects from prompts in Studies 1 and 2. First, in Study 3, the prompt was presented next to the ecological choice, as an indicator of the “greener” option. In Study 2, the prompt was a general message appearing at the top of the screen. The main difference was that, if participants would not have known which option was the most environment-friendly, in Study 3 they had the indication through the prompt, while in Study 2 they did not. While two-sided printing and recycled paper are evidently environment friendly, it is possible that some participants did not know that black/grayscale printing and reducing the quality level have an impact on the amount of ink used. However, we can surmise that this was not the case as we did not obtain a floor effect on those two choices. The other dimension on which the prompts in Study 3 differed from what was used in the other two studies was that it consisted of only a logo, with no verbal indication of the behavior (contrary to “Press on the button to turn off the computer screen” in Study 1 and “Please consider the environment while printing” in Study 2). This methodological choice was made due to the specificity of the task in Study 3: a logo felt more appropriate for a hypothetical choice task than a sentence asking participants to choose the most environment-friendly mode of transportation, which probably would have resulted in a ceiling effect since participants could have thought this was part of the task’s instructions.

## General Discussion

Widespread support for environmental protection is in many cases not followed by pro-environmental behavior. In this context, we tested experimentally how the use of prompts can make people with high levels of pro-environmental attitudes perform energy-saving behavior and hypothesized an interaction between those two variables. Three studies out of three did not support the interaction hypothesis: Study 1 did not allow for the testing of the hypothesis, Study 2 was inconclusive, and Study 3 supported the null. In light of our data, unmoderated main effects seem more plausible than an interaction. However, future studies need to confirm this claim.

### Implications

The present study contributes to the literature on the attitude-behavior link. Effect sizes of environmental attitudes ranged from Cohen’s *d* = 0.30 to *d* = 0.60. Those effect sizes are in the range of previous findings (Bamberg and Moser, 2007; Milfont, 2012) and confirm the association between pro-environmental attitudes and behavior.

This study also provides supporting data on the positive effect of prompts in eliciting pro-environmental behaviors (e.g., Sussman et al., 2013). Putting aside the impressive gain from 0 to 86% in Study 1, which might be an artifact of the experimental design as already discussed, effect sizes obtained for prompts ranged between Cohen’s *d* = 0.22 and *d* = 0.45. Those were slightly lower than the moderate-to-high effect sizes reported by Osbaldiston and Schott (2012) in their meta-analysis but confirmed that prompts can be a useful tool to trigger behavior (but see Sussman and Gifford, 2012, who showed that prompts are sometimes ineffective or in some cases even have a deleterious effect).

For attitudes, the biggest effect size was obtained in Study 3, while, for prompts, the biggest effect was found in Study 2. Differences in methods between studies prevented the claim that the size of the association between attitudes and behavior was stronger for mobility than energy savings and paper saving, and this also prevented the claim that the effect of prompts was bigger for paper saving than the other two studied behaviors (see, for example, the effect size homogeneity test in the study of Osbaldiston and Schott, 2012). Another option in the case of attitudes was that effect sizes were stronger for hypothetical choices than for real behavior (Chao and Lam, 2011; Kormos and Gifford, 2014).

Another point worth noting refers to the distinction observed between the specific dimensions of environmental attitudes. Our results showed that in Study 1, Preservation attitudes, compared to Utilization attitudes, were more strongly associated with environmental behavior, which replicates previous findings (Milfont and Duckitt, 2010; Milfont, 2012). Note, however, that in Study 2, the effect sizes of Preservation and Utilization were equivalents, and in Study 3, both dimensions were significantly associated with behavior. It is worth mentioning that the reliability of the Utilization sub-scale was consistently lower than reliability of the Preservation sub-scale, which might explain why the effect of Utilization was weaker in some cases.

While the three studies presented in this paper clearly supported the main effects of attitudes and prompts in predicting pro-environmental behavior, they did not support the hypothesis of an interaction, contrary to the interactionist perspective (Lewin, 1951) and the scarce empirical evidence (Wiese et al., 2004; Bolderdijk et al., 2013). Given the rather modest sizes of the Bayes factor obtained, we cannot completely rule out the idea that we simply did not detect the interaction; however, data suggested that the absence of the interaction was more likely. Interestingly, a similar conclusion was reached for the moderating role of behavioral difficulty in the attitude-behavior relationship by Kaiser and Schultz (2009); an interaction was expected, but data of several pooled studies showed that attitudes and difficulty were main effects but did not interact. The authors concluded that “the external conditions facilitate and impede behavior for all people similarly, like the slope of a mountain that is the same for everyone” (p. 203). More recently, Taube et al. (2018) have reported experimental support for the additive relationship between environmental attitudes and cost of the behavior but no interaction.

Recommendations for campaigns aiming at promoting pro-environmental behaviors would then be that prompts can be used as a “one-fits-all” technique. Similarly, enhancement of pro-environmental attitudes would be beneficial in terms of behavior, independently of the presence or absence of prompts in the environment of the person. It is important to mention that we obtained additive main effects, meaning that, if possible, both prompts and the enhancement of environmental attitudes should be combined to maximize behavior change, as we obtained across all three studies the most environment-friendly behaviors in the presence of prompts with participants with high levels of pro-environmental attitudes.

### Strengths and Limitations

One notable strength of two of our studies was that we had actual measures of behavior through the use of unobtrusive observation. There is a call for psychological research to measure behavior directly (Baumeister et al., 2007), and systematic observations of pro-environmental behavior have also been employed in recent environmental psychology studies (for a review, see Lange and Dewitte, 2019). We believe this is an important methodology for studies in the area (see Sussman, 2015). Although we observed behavior in a somewhat “unnatural” setting of a computer lab, this is a realistic and common behavior setting for university students and many people. Moreover, participants were not aware that their behavior was recorded, boosting the validity of our findings. Despite these strengths, in Study 2 a small number of participants did express suspicion about the real goal of the study and were thus excluded (5.33%, 8 of 150). Finding a task allowing observation of behavior without ambiguity for the participant has proven to be a challenge, and new developments in the field, such as the Pro-Environmental Behavior Task (Lange et al., 2018), are promising.

The fact that we measured environmental attitudes (instead of manipulating it) leads to caution in interpreting the effects observed for this variable. Because we did not manipulate attitudes, it can be argued that some other correlated variable might be responsible for the effect. What if we had measured other variables such as environmental values (see Bolderdijk et al., 2013) or personality traits (Milfont and Sibley, 2012)? Would the effect of those variables be stronger or different than the effect observed for environmental attitudes? From a conceptual point of view, environmental values are an antecedent of environmental attitudes (Schultz and Zelezny, 1999; Milfont et al., 2010), and the effect of personality traits on pro-environmental behavior has been shown to be mediated by environmental attitudes (Brick and Lewis, 2016; Busic-Sontic et al., 2017). Thus, it is possible that the effect of those variables is confounded with attitudes. Moreover, it seems particularly relevant to consider how individual characteristics other than attitudes do interact or not with prompts. Conversely, intention to perform the behavior is a more proximal determinant of behavior than attitudes. Would environmental intention to save electricity show stronger effects than attitudes and maybe interact with prompts? One might expect this to be the case. A prompt would probably not be enough to trigger behavior among people with no intention to act, while it is reasonable to expect the prompt to have an effect on people with high levels of intention. Indeed, the intention-behavior gap has sometimes been explained by forgetting the intention one has (Orbell et al., 1997), which might be resolved by prompts.

Our methodological choice to measure environmental attitudes at a general level and observe specific behaviors deserves discussion. According to Ajzen’s principle of measures compatibility (Ajzen, 2011), this should lessen the prediction power of attitudes. This is both a limitation and a strength, as we nonetheless observed statistically significant associations between attitudes and behavior. This suggests that effect sizes of attitudes-behavior relations might be underestimated in our studies and would have been bigger with measures of attitudes toward specific behaviors (i.e., attitude toward printing options, attitude toward mode of transportation, or attitude toward turning off the computer screen), and the effects of behavioral intention toward such behaviors could be even bigger.

The generalizability of the results was constrained by the reliance of university samples in all three studies. Convenience samples of undergraduate university students are not representative of the general population of the country and even less so of human beings worldwide (Henrich et al., 2010). Level of education, age, and gender distribution are example of variables that make our participants not representative. Notably, in all three studies, the proportion of men ranged between 16 and 28%. Importantly, levels of environmental attitudes have been shown to vary according to those variables (Gifford and Sussman, 2012; Milfont and Schultz, 2018). Thus, our participants might have been positively oriented toward pro-environmental action. Indeed, levels of environmental attitudes were above the scale’s middle point of 4 in all three samples^3^. However, we did not encounter any ceiling effect, which suggests that our results are interpretable.

### Future Studies

Other studies are needed to increase the amount of data on the important question of person-situation interaction in the environmental domain. One crucial element not addressed by our studies is the level of difficulty of the behavior. Although this was not central in our research question, it is possible that the interaction might occur at different levels of difficulty of the behavior. In our studies, all behaviors were either simple (Studies 1 and 2) or hypothetical (Study 3). However, we could imagine that for difficult or costly behavior, such as adopting a vegetarian diet every day, for example, prompts might have an effect only in people with high levels of environmental attitudes. Thus, in order to obtain a broader test of the attitudes-prompts interaction, several behaviors varying in their difficulty levels should be explored.

Other elements to take into account in future replications are notably the sample, which in our case was constituted only of university students. To increase the external validity of our results, targeting a broader and more diverse population would be recommended. Various types of prompts (e.g., Bergquist and Nilsson, 2016; Leoniak and Cwalina, 2019) could also be tested in interaction with attitudes, as the framing of a message could have a different impact depending on initial attitudes. Notably, the fact that the prompt only mentions the behavior (e.g., “Please turn off the lights”) versus refers to the environment as an argument to do the behavior (e.g., “Please turn off the lights to protect the environment”) does probably change the influence of attitudes, the former being even less susceptible to be influenced by the level of environmental attitudes of the person. Additionally, as mentioned in the limitations, other individual characteristics could be measured in future studies to compare their effects on behavior. Gifford and Nilsson (2014) reviewed 18 personal and social factors that influence pro-environmental behavior: childhood experience, knowledge and education, personality and self-construal, sense of control, values, political and world views, goals, felt responsibility, cognitive biases, place attachment, age, gender, chosen activities (i.e., personal factors), religion, urban-rural differences, norms, social class, proximity to problematic environmental sites, and cultural and ethnic variations (i.e., social factors). Considering those factors in future studies would allow to broaden the applicability of the results and would maybe lead to the further identification of moderators of the effect.

## Conclusion

Toward promoting individual environment-friendly behavior, attitudes and prompts are useful levers to pull. The individual effectiveness of each was known from past research, but this work has presented a rare investigation of their interplay and suggested that they appear to work independently of one another. Interventions aiming for maximum effectiveness should rely on both to trigger the most answers.

## Data Availability Statement

The datasets generated for this study are available on request to the corresponding author.

## Ethics Statement

The studies involving human participants were reviewed and approved by Human Ethics Committees at Victoria University (study 1) and Geneva University (studies 2 and 3). The patients/participants provided their written informed consent to participate in this study.

## Author Contributions

LM and TM conceived the first study, ran it, and analyzed the data. LM and OD conceived the second and third studies, ran them, and analyzed the data. The first version of the draft was written by LM and revised by TM and OD.

### Conflict of Interest

The authors declare that the research was conducted in the absence of any commercial or financial relationships that could be construed as a potential conflict of interest.

## Footnotes

^1^We needed to counterbalance which ones where associated with the prompt or not to prevent a confounding effect due to characteristics of the destinations (i.e. distance, accessibility, etc.).

^2^A possible explanation for this result is that Venice is the farthest destination among the four scenarios, and thus, unless it is associated with the prompt, nobody considered going there other than with the plane.

^3^We calculated one-sample *t*-tests for each study. Study 1 (*N* = 185; *M* = 4.74, *SD* = 0.66): *t* (184) = 15.25, *p* < 0.001, *d* = 1.12. Study 2 (*N* = 150; *M* = 4.88, *SD* = 0.56): *t* (149) = 19.25, *p* < 0.001, *d* = 1.57. Study 3 (*N* = 180; *M* = 5.01, *SD* = 0.69): *t* (179) = 19.64, *p* < 0.001, *d* = 1.46.
