# Supplementary material for: Do Environmental Prompts Work the Same for Everyone? A Test of Environmental Attitudes as a Moderator
Source: Front Psychol. 2020 Feb 5;10:3057. doi: 10.3389/fpsyg.2019.03057 (PMC7015073; doi:10.3389/fpsyg.2019.03057)
Supplement: Supplementary file 1 [file Data_Sheet_1.docx]

Supplementary Materials

# Study 1 material


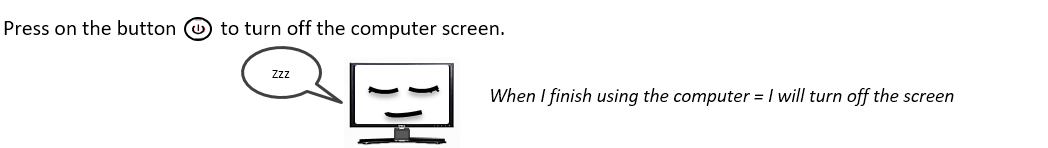


Figure S1. Message-only prompt used in Study 1.


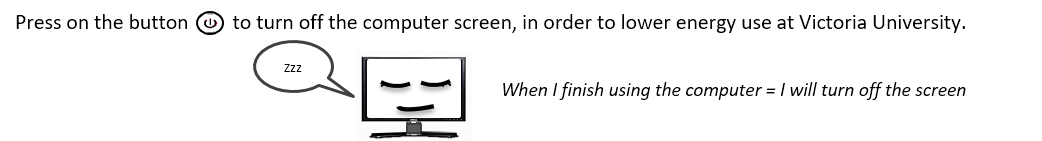


Figure S2. Low-level goal prompt used in Study 1.


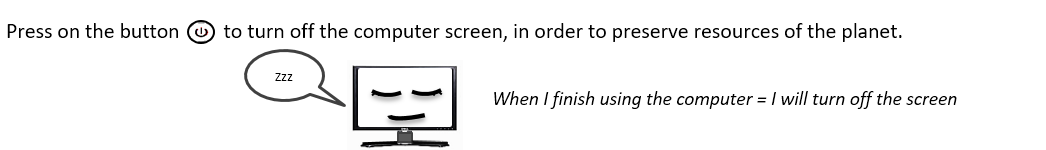


Figure S3. High-level goal prompt used in Study 1.

# Study 2 material


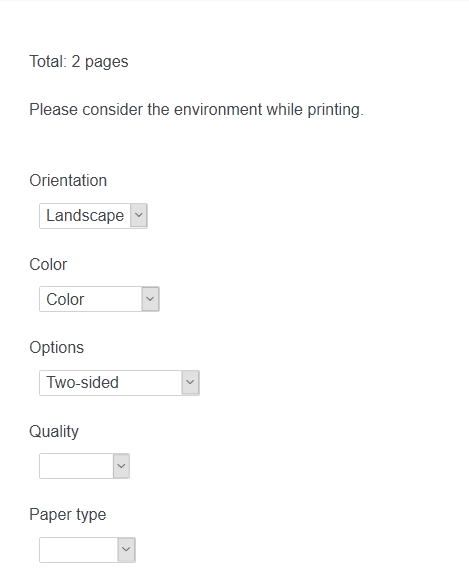

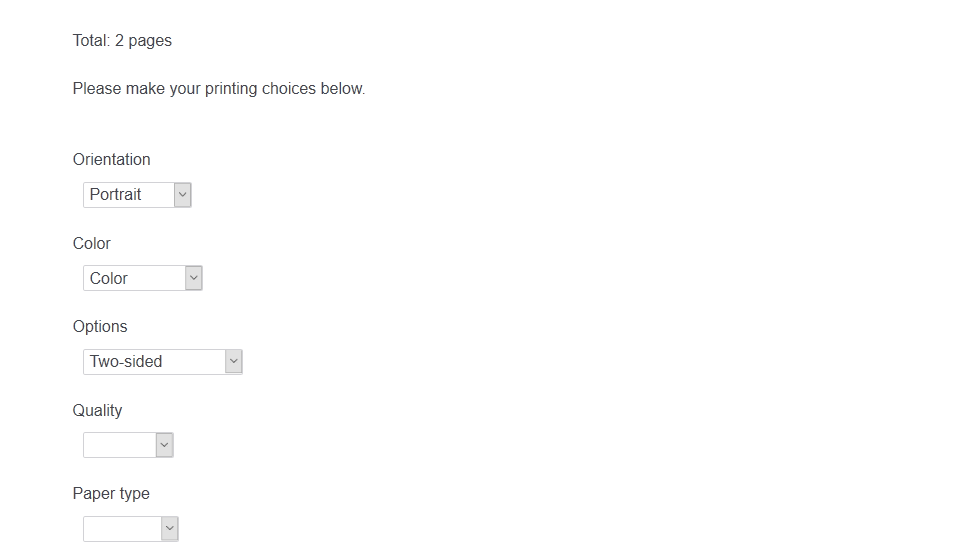


Figure S4. Screenshot of the printing preference page used in Study 2 (with examples of printing preferences selected). Left: no-prompt condition. Right: prompt condition.

# Study 2 supplementary information

Regarding the exclusion of participants that expressed suspicion about the printing task, we considered it was better to exclude participants that did not believed the stated goal of the experiment because we thought their answer on the printing choices would not be genuine. The link with the mechanisms examined is that they might have been more suspicious in the “prompt” condition, and their reactions might differ according to their level of attitude (maybe participants with high levels of attitudes exaggerate the “green” choices when they know their choice is recorded, while participants with low levels of attitudes would have a “reactance”-type of reaction and choose the less “green” options). Suspicion was less frequent in the no prompt condition, which we believe highlight the possible problem, compared as if it was “randomly distributed” among conditions.

# Study 3 material

Hypothetical choices of transportation mode used in Study 3. The green signs are the prompts.

[vignette1] Imagine that you are planning to go on holiday to Venice for 1 week. Which mode of transport would you choose?
-train (about 7 hours)
-plane (1h15 flight plus time to complete the formalities at the airport)

[filler] Imagine that you have to go to rue du Rhône in downtown Geneva for a job interview. Which mode of transport would you choose?
-by foot
-public transport


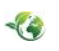
[vignette2] Imagine that you have to go to Ticino for a professional event with a night on site. Which mode of transport would you choose?
-train (about 6 hours)
-plane (45 minutes flight plus time to complete the formalities at the airport)

[vignette3] Imagine that you have to go to Paris for the weekend, and that you can only leave on Friday at the end of the day and have to return on Sunday afternoon. Which mode of transport would you choose?
-plane (1 hour flight plus time to complete the formalities at the airport)
-TGV (3 hours)

[filler] Imagine that you want to go to Australia for several months. Which transport option would you choose?
-plane, direct flight
-plane, 1 stopover


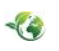
[vignette4] Imagine that you have to go to Lausanne for the day, arrive on site at about 7:30 am and return around 8 pm. Which mode of transport would you choose?
-train (between 30 and 50 minutes)
-car (50 minutes) (if you had a car)

# Study 3 supplementary results


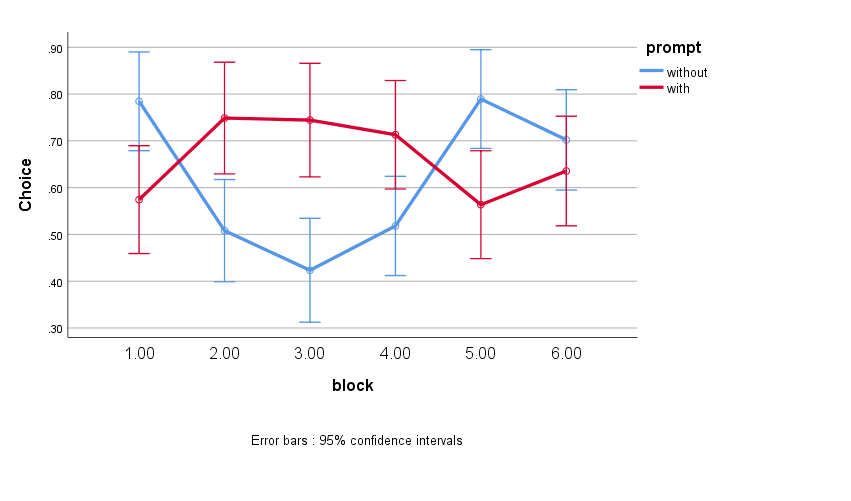


Figure S5: *Interaction between the prompt condition (presence/absence) and the block on the dependent variable assessing choice of transportation mode (the higher the value, the greater the choice for the environment-friendly mode of transportation).*Note: in each block the order of presentation of the scenarios was random and mixed with two fillers

Block 1: *With prompt:* Venice and Ticino / *Without prompt:* Paris and Lausanne

Block 2: *With prompt:* Paris and Lausanne / *Without prompt:* Venice and Ticino

Block 3: *With prompt:* Paris and Ticino / *Without prompt:* Venice and Lausanne

Block 4: *With prompt:* Lausanne and Ticino / *Without prompt:* Venice and Paris

Block 5: *With prompt:* Venice and Lausanne / *Without prompt:* Paris and Ticino

Block 6: *With prompt:* Venice and Paris / *Without prompt:* Lausanne and Ticino
